# Prehospital Detection of Large Vessel Occlusion Stroke With EEG: Results of the ELECTRA-STROKE Study

**DOI:** 10.1212/WNL.0000000000207831

**Published:** 2023-12-12

**Authors:** Maritta N. van Stigt, Eva A. Groenendijk, Laura C.C. van Meenen, Anita A.G.A. van de Munckhof, Monique Theunissen, Gaby Franschman, Martin D. Smeekes, Joffry A.F. van Grondelle, Geertje Geuzebroek, Arjen Siegers, Marieke C. Visser, Sander M. van Schaik, Patricia H.A. Halkes, Charles B.L.M. Majoie, Yvo B.W.E.M. Roos, Johannes H.T.M. Koelman, Miou S. Koopman, Henk A. Marquering, Wouter V. Potters, Jonathan M. Coutinho

**Affiliations:** From the Departments of Clinical Neurophysiology (M.N.v.S., E.A.G., J.H.T.M.K.), Neurology (M.N.v.S., E.A.G., L.C.C.v.M., A.A.G.A.v.d.M., M.C.V., Y.B.W.E.M.R., J.M.C.), Radiology and Nuclear Medicine (C.B.L.M.M., M.S.K., H.A.M.), and Biomedical Engineering and Physics (H.A.M.), Amsterdam UMC location University of Amsterdam; Witte Kruis Ambulancezorg (M.T., G.F.), Alkmaar; Ambulancezorg Nederland (M.D.S.), Zwolle; Ambulance Amsterdam (J.A.F.v.G., G.G., A.S.); Department of Neurology (S.M.v.S.), OLVG Hospital location West, Amsterdam; Department of Neurology (P.H.A.H.), Noordwest Ziekenhuisgroep location Alkmaar; TrianecT (W.V.P.), Utrecht, the Netherlands.

## Abstract

**Background and Objectives:**

Endovascular thrombectomy (EVT) is standard treatment for anterior large vessel occlusion stroke (LVO-a stroke). Prehospital diagnosis of LVO-a stroke would reduce time to EVT by allowing direct transportation to an EVT-capable hospital. We aim to evaluate the diagnostic accuracy of dry electrode EEG for the detection of LVO-a stroke in the prehospital setting.

**Methods:**

ELECTRA-STROKE was an investigator-initiated, prospective, multicenter, diagnostic study, performed in the prehospital setting. Adult patients were eligible if they had suspected stroke (as assessed by the attending ambulance nurse) and symptom onset <24 hours. A single dry electrode EEG recording (8 electrodes) was performed by ambulance personnel. Primary endpoint was the diagnostic accuracy of the theta/alpha frequency ratio for LVO-a stroke (intracranial ICA, A1, M1, or proximal M2 occlusion) detection among patients with EEG data of sufficient quality, expressed as the area under the receiver operating characteristic curve (AUC). Secondary endpoints were diagnostic accuracies of other EEG features quantifying frequency band power and the pairwise derived Brain Symmetry Index. Neuroimaging was assessed by a neuroradiologist blinded to EEG results.

**Results:**

Between August 2020 and September 2022, 311 patients were included. The median EEG duration time was 151 (interquartile range [IQR] 151–152) seconds. For 212/311 (68%) patients, EEG data were of sufficient quality for analysis. The median age was 74 (IQR 66–81) years, 90/212 (42%) were women, and the median baseline NIH Stroke Scale was 1 (IQR 0–4). Six (3%) patients had an LVO-a stroke, 109/212 (51%) had a non–LVO-a ischemic stroke, 32/212 (15%) had a transient ischemic attack, 8/212 (4%) had a hemorrhagic stroke, and 57/212 (27%) had a stroke mimic. AUC of the theta/alpha ratio was 0.80 (95% CI 0.58–1.00). Of the secondary endpoints, the pairwise derived Brain Symmetry Index in the delta frequency band had the highest diagnostic accuracy (AUC 0.91 [95% CI 0.73–1.00], sensitivity 80% [95% CI 38%–96%], specificity 93% [95% CI 88%–96%], positive likelihood ratio 11.0 [95% CI 5.5–21.7]).

**Discussion:**

The data from this study suggest that dry electrode EEG has the potential to detect LVO-a stroke among patients with suspected stroke in the prehospital setting. Toward future implementation of EEG in prehospital stroke care, EEG data quality needs to be improved.

**Trial Registration Information:**

ClinicalTrials.gov identifier: NCT03699397.

**Classification of Evidence:**

This study provides Class II evidence that prehospital dry electrode scalp EEG accurately detects LVO-a stroke among patients with suspected acute stroke.

## Introduction

Endovascular thrombectomy (EVT) is standard treatment for patients with an anterior circulation large vessel occlusion stroke (LVO-a stroke).^1^ EVT must be initiated as soon as possible because patient outcome after EVT is highly time-dependent.^2-4^ A large group of LVO-a stroke patients (45%–83%) is initially transported to a non–EVT-capable primary stroke center, necessitating interhospital transfer to a comprehensive stroke center.^5-9^ On average, this so-called drip-and-ship workflow delays the initiation of EVT by 39–114 minutes and is associated with a 7.8%–21.4% lower chance of functional independence at 90 days.^5,7,9,10,11^

A triage instrument that can quickly and reliably identify patients with an LVO-a stroke in the prehospital setting would allow direct transportation of these patients to a comprehensive stroke center, which could substantially shorten EVT treatment initiation times. Several methods for prehospital detection of LVO-a stroke currently exist, including clinical stroke scales and mobile stroke units.^12-18^ However, none of these methods have been implemented on a widespread scale, and inadequate prehospital stroke triage remains an important issue in acute care organization. EEG is a promising technique for prehospital stroke triage because it is highly sensitive to the reduction of the cerebral blood flow almost immediately after onset.^19-23^ Previous studies have shown that EEG can discriminate between LVO-a stroke patients and other suspected stroke patients in an in-hospital setting,^24,25,26^ but studies in the prehospital setting have not been performed. The primary aim of the ELECTRA-STROKE study was to determine the diagnostic accuracy of dry electrode EEG for LVO-a stroke detection in the prehospital setting, expressed as the area under the receiver operating characteristic (ROC) curve (AUC) of the theta/alpha ratio.

## Methods

### Study Design and Population

ELECTRA-STROKE was a prospective, investigator-initiated, multicenter, diagnostic study, consisting of an in-hospital and a prehospital phase. The results of the in-hospital phase have been published previously.^26^ Here, we report the results of the prehospital phase.

The study was performed by 2 ambulance services (Ambulance Amsterdam and Witte Kruis Ambulancezorg Alkmaar) and 3 receiving hospitals in the Dutch province of North Holland (Amsterdam UMC, OLVG Hospital, and Noordwest Ziekenhuisgroep). The patient recruitment took place between August 2020 and September 2022.

We included adult patients with a clinically suspected stroke as judged by the ambulance nurse and onset of symptoms or time last seen well less than 24 hours before start of the EEG recording. Patients with a wound or active infection of the scalp in the dry electrode cap placement area and patients with a (suspected) COVID-19 infection were excluded.

### Standard Protocol Approvals, Registrations, and Patient Consents

The study protocol (eSAP, links.lww.com/WNL/D121) was approved by the institutional review board of the Amsterdam UMC and by each participating hospital. The study protocol was published previously.^27^ The study was monitored by the Clinical Research Unit of the Amsterdam UMC. Because of the emergency setting of the study, we used a deferred informed consent procedure in line with national legislation and with approval of the institutional review board.^28^ Written informed consent was obtained from all patients or their legal representatives after EEG data acquisition.

### Study Procedures

**Figure 1:**
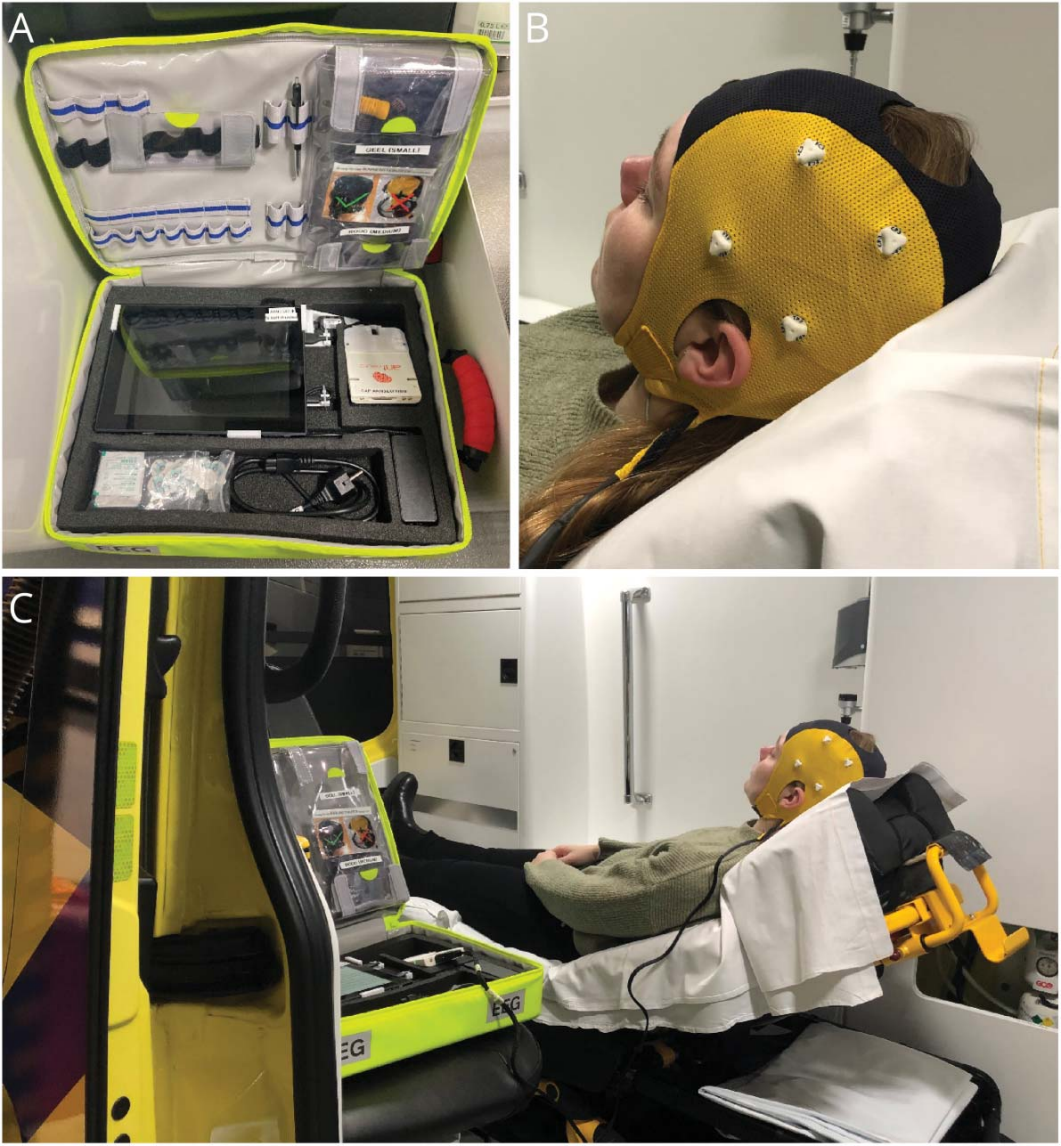
EEG Setup as Used in the ELECTRA-STROKE Study (A) EEG equipment stored in a portable and lightweight bag. (B) Dry electrode EEG cap (Waveguard touch, Eemagine, Berlin, Germany). (C) EEG recording in the ambulance.

A single EEG recording was performed using a dry electrode EEG cap with 8 electrodes covering the vascular territory of the middle cerebral artery (FC3, FC4, CP3, CP4, FT7, FT8, TP7, and TP8; Waveguard touch, Eemagine, Berlin, Germany) and a compatible EEG amplifier (eego amplifier EE-411; Eemagine, Berlin, Germany). EEG data were acquired at a sample frequency of 500 Hz using clinical EEG software (NeuroCenter EEG, Clinical Science Systems, Leiden, The Netherlands). EEG recordings were performed in the prehospital setting by ambulance personnel who attended a 1-hour training in performing dry electrode EEG recordings for the purpose of this study. A portable and lightweight bag was used to store all equipment required for the EEG recording (Figure 1). In December 2020 (Witte Kruis Ambulancezorg Alkmaar) and February 2021 (Ambulance Amsterdam), we adapted the acquisition software to provide visual feedback on the electrode-skin impedances to guide optimization of the electrode-skin contact. We time-limited both the impedance optimization step (1.5 minutes) and the EEG recording step (2.5–3.0 minutes) to minimize delay in the prehospital workflow. As of January 2021, we collected information on the length of the hair of the patients in whom an EEG recording was performed by ambulance personnel of Witte Kruis Ambulancezorg Alkmaar.

Routine stroke workup included a noncontrast CT, and, if indicated, CT angiography and CT perfusion were performed in the receiving hospital.

### Definitions and Outcomes

The primary endpoint of the study was the diagnostic accuracy of dry electrode EEG to discriminate LVO-a stroke from all other strokes and stroke mimics, expressed as the AUC of the theta/alpha ratio. The theta/alpha ratio was defined as:
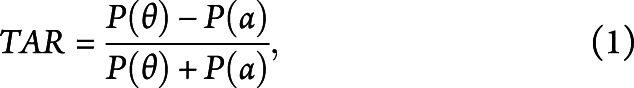
with 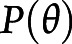 the power in the theta frequency band (4–8 Hz) and 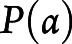 the power in the alpha frequency band (8–13 Hz). The theta/alpha ratio was chosen as this EEG feature had highest diagnostic accuracy for LVO-a stroke detection in the first 100 patients included in the in-hospital phase of the study.^26^ Besides the AUC, sensitivity, specificity, positive predictive value, negative predictive value, and positive likelihood ratio were determined.

Secondary endpoints, which should be considered exploratory only, were the diagnostic accuracies of the relative delta (1–4 Hz), theta (4–8 Hz), alpha (8–13 Hz), and beta (13–18 Hz) power; the delta/alpha ratio, the (delta + theta)/(alpha + beta) ratio, and the pairwise derived Brain Symmetry Index for LVO-a stroke detection; and the logistical and technical feasibility of ambulance personnel performing an EEG recording with a dry electrode cap in the prehospital setting in patients with a suspected stroke. Definitions of the EEG features are included in the eAppendix 1 (links.lww.com/WNL/D119). We quantified the logistical and technical feasibility by calculating the percentage of patients with EEG data of sufficient quality for analysis and the percentage of clean EEG data per patient. To account for learning effects and hardware and software improvements, we compared the percentage of clean EEG data of the first 50 EEG recordings with the percentage of clean EEG data of the last 50 EEG recordings performed in this study. The principal safety outcome was the occurrence of device-related adverse events.

We defined an LVO-a stroke as an occlusion of the intracranial part of the internal carotid artery, the first or proximal second segment of the middle cerebral artery (M1 and proximal M2, respectively), or the first segment of the anterior cerebral artery (A1). The occlusion location was determined by an adjudication committee—consisting of a neuroradiologist (M.S.K.) and a stroke neurologist (J.M.C.)—based on CT angiography data. The adjudication committee was blinded for EEG results. In case a patient was not clinically suspected of having an LVO-a stroke and therefore did not undergo CT angiography, the patient was scored as not having an LVO-a stroke.

### Data Analysis

EEG data were re-referenced to a 12-channel bipolar montage with 6 bipolar channels located at each hemisphere and bandpass filtered between 0.5 and 35 Hz using a third order Butterworth filter. Artifacts were detected and rejected per 2-second bipolar channel segment using an automatic Convolutional Neural Network algorithm specifically developed for the detection of artifacts in dry electrode EEG data.^29^ The pairwise derived Brain Symmetry Index was calculated using EEG channels located on both hemispheres. All other EEG features were calculated per hemisphere. For patients with a stroke, we kept the ipsilesional EEG features, and for patients with a stroke mimic, we averaged the EEG features over both hemispheres (if available). Further details are provided in the eMethods (links.lww.com/WNL/D120).

### Statistical Analysis

The sample size was calculated based on an expected specificity of dry electrode EEG for LVO-a stroke of 70%. At the start of the study, the sample size was set at 222, based on an expected incidence of LVO-a stroke of 7% and a dropout rate of 20%. An interim analysis, based on 137 suspected stroke patients in whom a dry electrode EEG recording was performed in the prehospital setting, showed an actual incidence of LVO-a stroke of 5% and a dropout rate of 58%. Patients were considered a dropout if EEG data were of insufficient quality, time of symptom onset was more than 24 hours before the start of the EEG recording, or no informed consent was provided. A revised incidence of LVO-a stroke of 5%, an alpha of 0.05 and a maximum margin of error of 7% indicated that 174 patients with a suspected stroke were required.^30^ As EEG data quality improved over time,^26^ we assumed a dropout rate of 55% when recalculating the sample size. Considering this dropout rate, our revised estimation resulted in a sample size of 386 patients with a suspected stroke.

The diagnostic accuracies of all EEG features were evaluated by calculating areas under the ROC curves. Two cutoff values were determined as the highest sensitivity at a specificity of ≥70% and ≥80% for LVO-a stroke, respectively. The latter is defined as optimal cutoff. For both cutoff values, sensitivity, specificity, positive predictive value, negative predictive value, and positive likelihood ratio are reported with 95% confidence intervals. The 95% confidence intervals were estimated using the method of Hanley and McNeil^31^ for the AUC; the Wilson interval^32^ for the sensitivity, specificity, positive predictive value, and negative predictive value; and the method of Simel et al.^33^ for the positive likelihood ratio.

Baseline characteristics and EEG feature values of patients with an LVO-a stroke and those without an LVO-a stroke were compared using the independent samples *t* test for normally distributed continuous variables, the Mann-Whitney *U* test for non-normally distributed continuous variables, and the χ^2^ test (if any of the cells had expected count ≥5) or Fisher exact test (if any of the cells had expected count <5) for categorical variables. Percentages of clean EEG data of the first and last 50 EEG recordings were compared using the Mann-Whitney *U* test. A *p* value ≤0.05 was considered statistically significant.

Data were analyzed with Python (version 3.8; Python Software Foundation, Wilmington, DE).

### Data Availability

Individual patient data cannot be made available under Dutch law because we did not obtain patient approval for sharing individual patient data, even in coded form.

## Results

**Figure 2:**
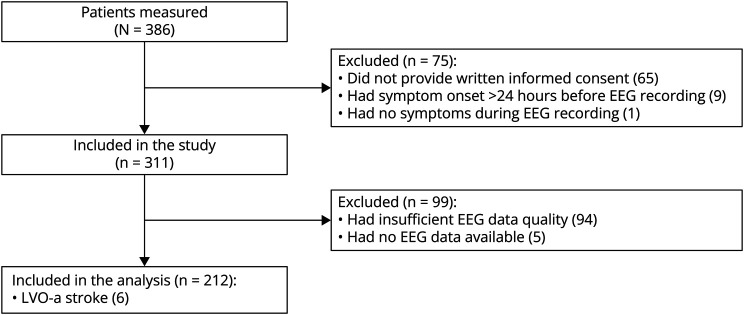
Patient Flowchart LVO-a stroke = anterior circulation large vessel occlusion stroke.

Ambulance personnel performed a dry electrode EEG recording in 386 patients, of whom 75/386 (19%) were subsequently excluded because no informed consent could be obtained (n = 65), symptom onset was >24 hours (n = 9), or symptoms were absent during EEG recording (n = 1; Figure 2). Of the 311 patients included in the study, 99 (32%) were excluded from analysis because of insufficient quality of the EEG data (n = 94) or the absence of EEG data due to technical failure (n = 5). The median (interquartile range [IQR]) percentage of clean EEG data over all patients was 14% (4%–39%). Data quality improved over time (median [IQR] percentage of clean EEG data of the first 50 EEG recordings vs the last 50 EEG recordings: 11% [9%–12%] vs 26% [25%–27%], *p* < 0.001). The median EEG duration time was 151 (IQR 151–152) seconds. A comparison of baseline characteristics between patients included and excluded in the analysis is presented in Table 1. Excluded patients were more often female (59% vs 42%, *p* = 0.01) and more often had an LVO-a stroke (9% vs 3%, *p* = 0.02). Patients who were excluded from analysis also more often had long hair (32% vs 15%, *p* = 0.07).

**Table 1:**
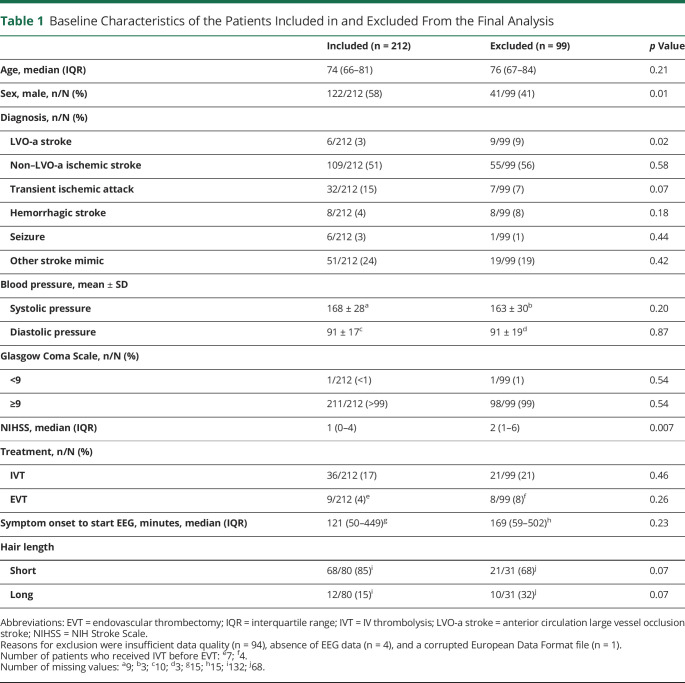
Baseline Characteristics of the Patients Included in and Excluded From the Final Analysis

	Included (n = 212)	Excluded (n = 99)	*p* Value
Age, median (IQR)	74 (66–81)	76 (67–84)	0.21
Sex, male, n/N (%)	122/212 (58)	41/99 (41)	0.01
Diagnosis, n/N (%)			
LVO-a stroke	6/212 (3)	9/99 (9)	0.02
Non–LVO-a ischemic stroke	109/212 (51)	55/99 (56)	0.58
Transient ischemic attack	32/212 (15)	7/99 (7)	0.07
Hemorrhagic stroke	8/212 (4)	8/99 (8)	0.18
Seizure	6/212 (3)	1/99 (1)	0.44
Other stroke mimic	51/212 (24)	19/99 (19)	0.42
Blood pressure, mean ± SD			
Systolic pressure	168 ± 28^a^	163 ± 30^b^	0.20
Diastolic pressure	91 ± 17^c^	91 ± 19^d^	0.87
Glasgow Coma Scale, n/N (%)			
<9	1/212 (<1)	1/99 (1)	0.54
≥9	211/212 (>99)	98/99 (99)	0.54
NIHSS, median (IQR)	1 (0–4)	2 (1–6)	0.007
Treatment, n/N (%)			
IVT	36/212 (17)	21/99 (21)	0.46
EVT	9/212 (4)^e^	8/99 (8)^f^	0.26
Symptom onset to start EEG, minutes, median (IQR)	121 (50–449)^g^	169 (59–502)^h^	0.23
Hair length			
Short	68/80 (85)^i^	21/31 (68)^j^	0.07
Long	12/80 (15)^i^	10/31 (32)^j^	0.07

Abbreviations: EVT = endovascular thrombectomy; IQR = interquartile range; IVT = IV thrombolysis; LVO-a stroke = anterior circulation large vessel occlusion stroke; NIHSS = NIH Stroke Scale.

Reasons for exclusion were insufficient data quality (n = 94), absence of EEG data (n = 4), and a corrupted European Data Format file (n = 1).

Number of patients who received IVT before EVT: ^e^7; ^f^4.

Number of missing values: ^a^9; ^b^3; ^c^10; ^d^3; ^g^15; ^h^15; ^i^132; ^j^68.

Of the 212 patients included in data analysis, 6 (3%) had an LVO-a stroke, 109 (51%) a non–LVO-a ischemic stroke, 32 (15%) a TIA, 8 (4%) a hemorrhagic stroke, and 57 (27%) a stroke mimic. Patients with an LVO-a stroke had a higher median NIH Stroke Scale (15 vs 1, *p* < 0.001), more often received IV thrombolysis and EVT (67% vs 16%, *p* = 0.008 and 83% vs 2%, *p* < 0.001, respectively), and had lower onset to EEG time (median: 46 vs 139 minutes, *p* = 0.05) (Table 2). EEG recordings were performed before initiation of any reperfusion therapy in all patients. No device-related adverse events were reported.

**Table 2:**
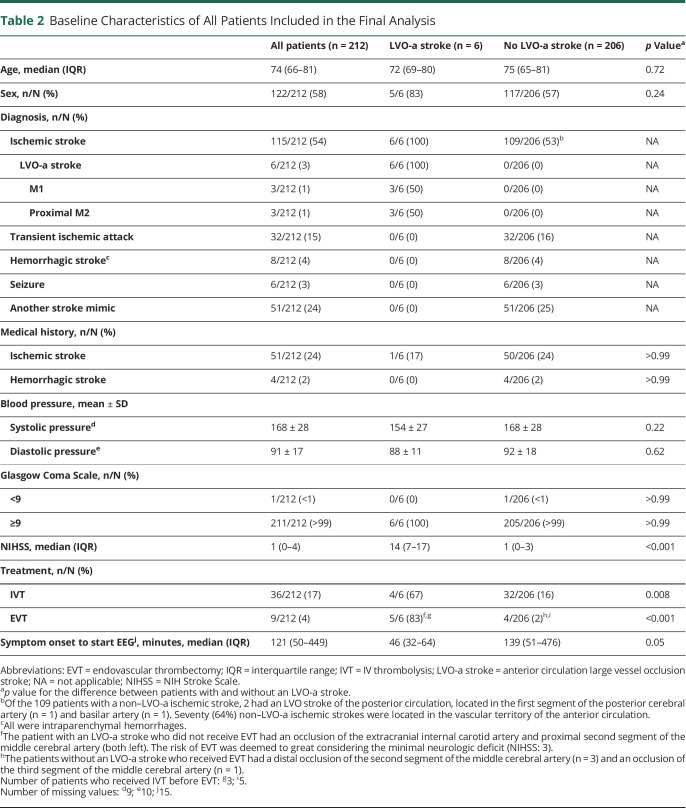
Baseline Characteristics of All Patients Included in the Final Analysis

	All patients (n = 212)	LVO-a stroke (n = 6)	No LVO-a stroke (n = 206)	*p* Value^a^
Age, median (IQR)	74 (66–81)	72 (69–80)	75 (65–81)	0.72
Sex, n/N (%)	122/212 (58)	5/6 (83)	117/206 (57)	0.24
Diagnosis, n/N (%)				
Ischemic stroke	115/212 (54)	6/6 (100)	109/206 (53)^b^	NA
LVO-a stroke	6/212 (3)	6/6 (100)	0/206 (0)	NA
M1	3/212 (1)	3/6 (50)	0/206 (0)	NA
Proximal M2	3/212 (1)	3/6 (50)	0/206 (0)	NA
Transient ischemic attack	32/212 (15)	0/6 (0)	32/206 (16)	NA
Hemorrhagic stroke^c^	8/212 (4)	0/6 (0)	8/206 (4)	NA
Seizure	6/212 (3)	0/6 (0)	6/206 (3)	NA
Another stroke mimic	51/212 (24)	0/6 (0)	51/206 (25)	NA
Medical history, n/N (%)				
Ischemic stroke	51/212 (24)	1/6 (17)	50/206 (24)	>0.99
Hemorrhagic stroke	4/212 (2)	0/6 (0)	4/206 (2)	>0.99
Blood pressure, mean ± SD				
Systolic pressure^d^	168 ± 28	154 ± 27	168 ± 28	0.22
Diastolic pressure^e^	91 ± 17	88 ± 11	92 ± 18	0.62
Glasgow Coma Scale, n/N (%)				
<9	1/212 (<1)	0/6 (0)	1/206 (<1)	>0.99
≥9	211/212 (>99)	6/6 (100)	205/206 (>99)	>0.99
NIHSS, median (IQR)	1 (0–4)	14 (7–17)	1 (0–3)	<0.001
Treatment, n/N (%)				
IVT	36/212 (17)	4/6 (67)	32/206 (16)	0.008
EVT	9/212 (4)	5/6 (83)^f,g^	4/206 (2)^h,i^	<0.001
Symptom onset to start EEG^j^, minutes, median (IQR)	121 (50–449)	46 (32–64)	139 (51–476)	0.05

Abbreviations: EVT = endovascular thrombectomy; IQR = interquartile range; IVT = IV thrombolysis; LVO-a stroke = anterior circulation large vessel occlusion stroke; NA = not applicable; NIHSS = NIH Stroke Scale.

^a^*p* value for the difference between patients with and without an LVO-a stroke.

^b^Of the 109 patients with a non–LVO-a ischemic stroke, 2 had an LVO stroke of the posterior circulation, located in the first segment of the posterior cerebral artery (n = 1) and basilar artery (n = 1). Seventy (64%) non–LVO-a ischemic strokes were located in the vascular territory of the anterior circulation.

^c^All were intraparenchymal hemorrhages.

^f^The patient with an LVO-a stroke who did not receive EVT had an occlusion of the extracranial internal carotid artery and proximal second segment of the middle cerebral artery (both left). The risk of EVT was deemed to great considering the minimal neurologic deficit (NIHSS: 3).

^h^The patients without an LVO-a stroke who received EVT had a distal occlusion of the second segment of the middle cerebral artery (n = 3) and an occlusion of the third segment of the middle cerebral artery (n = 1).

Number of patients who received IVT before EVT: ^g^3; ^i^5.

Number of missing values: ^d^9; ^e^10; ^j^15.

**Figure 3:**
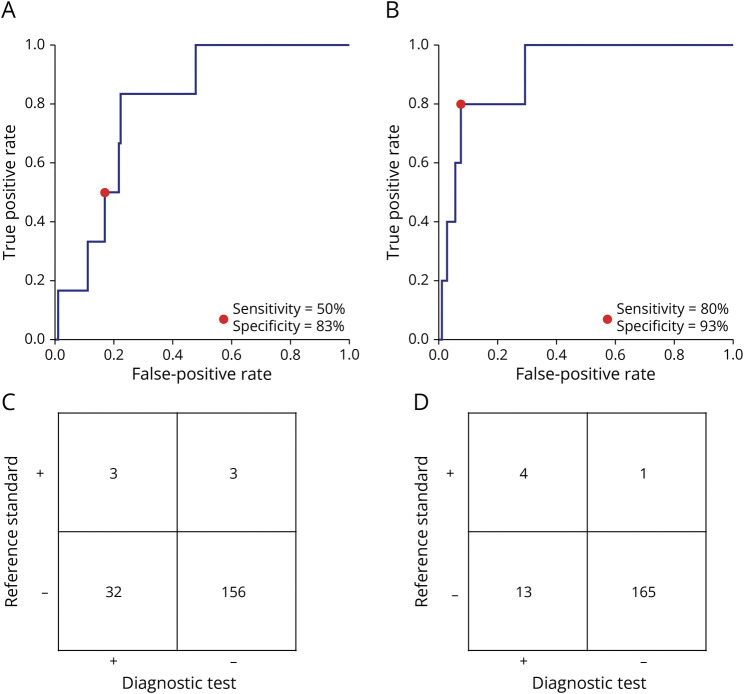
ROC Curves for LVO-a Stroke Detection (A) ROC curve of the theta/alpha ratio with, at optimal cutoff, a sensitivity of 50% and specificity of 83% for LVO-a stroke. (B) ROC curve of the pairwise derived Brain Symmetry Index in the delta frequency band with, at optimal cutoff, a sensitivity of 80% and specificity of 93% for LVO-a stroke. (C) Contingency table of LVO-a stroke prediction by the theta/alpha ratio. (D) Contingency table of LVO-a stroke prediction by the pairwise derived Brain Symmetry Index in the delta frequency band. LVO-a stroke = anterior circulation large vessel occlusion stroke; ROC = receiver operating characteristic.

For the frequency band powers and pairwise derived Brain Symmetry Index, 194 (6 LVO-a stroke) and 183 (5 LVO-a stroke) patients were available for analysis, respectively. The AUC of the theta/alpha ratio was 0.80 (95% CI 0.58–1.00; Table 3; Figure 3A). At optimal cutoff, we found a sensitivity of 50% (95% CI 19%–81%), a specificity of 83% (95% CI 77%–88%), a positive predictive value of 9% (95% CI 3%–22%), and a negative predictive value of 98% (95% CI 95%–99%; Figure 3C). Of the secondary endpoints, the pairwise derived Brain Symmetry Index in the delta frequency band had highest diagnostic accuracy for LVO-a stroke detection with an AUC of 0.91 (95% CI 0.73–1.00); at optimal cutoff, a sensitivity of 80% (95% CI 38%–96%) and a specificity of 93% (95% CI 88%–96%) (Figure 3, B and D). The diagnostic measures of all EEG features at maximum sensitivity at a specificity of ≥70% are presented in eTable 1 (links.lww.com/WNL/D122). The EEG feature values for patients with an LVO-a stroke and patients without an LVO-a stroke are presented in eTable 2 (links.lww.com/WNL/D123).

**Table 3:**
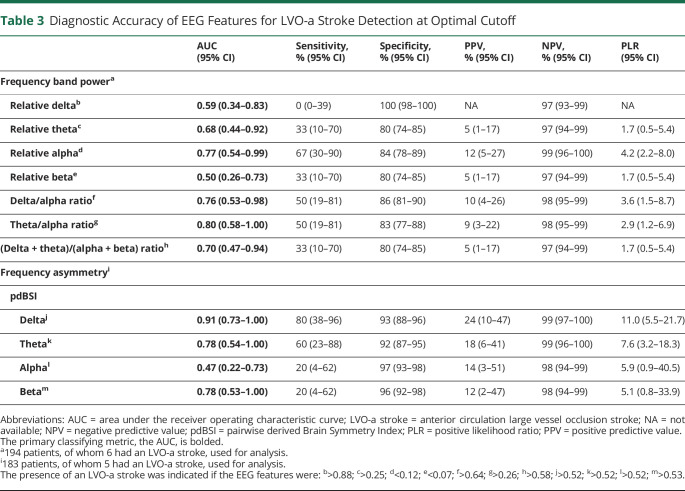
Diagnostic Accuracy of EEG Features for LVO-a Stroke Detection at Optimal Cutoff

	AUC (95% CI)	Sensitivity, % (95% CI)	Specificity, % (95% CI)	PPV, % (95% CI)	NPV, % (95% CI)	PLR (95% CI)
Frequency band power^a^						
Relative delta^b^	**0.59 (0.34–0.83)**	0 (0–39)	100 (98–100)	NA	97 (93–99)	NA
Relative theta^c^	**0.68 (0.44–0.92)**	33 (10–70)	80 (74–85)	5 (1–17)	97 (94–99)	1.7 (0.5–5.4)
Relative alpha^d^	**0.77 (0.54–0.99)**	67 (30–90)	84 (78–89)	12 (5–27)	99 (96–100)	4.2 (2.2–8.0)
Relative beta^e^	**0.50 (0.26–0.73)**	33 (10–70)	80 (74–85)	5 (1–17)	97 (94–99)	1.7 (0.5–5.4)
Delta/alpha ratio^f^	**0.76 (0.53–0.98)**	50 (19–81)	86 (81–90)	10 (4–26)	98 (95–99)	3.6 (1.5–8.7)
Theta/alpha ratio^g^	**0.80 (0.58–1.00)**	50 (19–81)	83 (77–88)	9 (3–22)	98 (95–99)	2.9 (1.2–6.9)
(Delta + theta)/(alpha + beta) ratio^h^	**0.70 (0.47–0.94)**	33 (10–70)	80 (74–85)	5 (1–17)	97 (94–99)	1.7 (0.5–5.4)
Frequency asymmetry^i^						
pdBSI						
Delta^j^	**0.91 (0.73–1.00)**	80 (38–96)	93 (88–96)	24 (10–47)	99 (97–100)	11.0 (5.5–21.7)
Theta^k^	**0.78 (0.54–1.00)**	60 (23–88)	92 (87–95)	18 (6–41)	99 (96–100)	7.6 (3.2–18.3)
Alpha^l^	**0.47 (0.22–0.73)**	20 (4–62)	97 (93–98)	14 (3–51)	98 (94–99)	5.9 (0.9–40.5)
Beta^m^	**0.78 (0.53–1.00)**	20 (4–62)	96 (92–98)	12 (2–47)	98 (94–99)	5.1 (0.8–33.9)

Abbreviations: AUC = area under the receiver operating characteristic curve; LVO-a stroke = anterior circulation large vessel occlusion stroke; NA = not available; NPV = negative predictive value; pdBSI = pairwise derived Brain Symmetry Index; PLR = positive likelihood ratio; PPV = positive predictive value.

The primary classifying metric, the AUC, is bolded.

^a^194 patients, of whom 6 had an LVO-a stroke, used for analysis.

^i^183 patients, of whom 5 had an LVO-a stroke, used for analysis.

The presence of an LVO-a stroke was indicated if the EEG features were: ^b^>0.88; ^c^>0.25; ^d^<0.12; ^e^<0.07; ^f^>0.64; ^g^>0.26; ^h^>0.58; ^j^>0.52; ^k^>0.52; ^l^>0.52; ^m^>0.53.

## Discussion

In this prehospital study, dry electrode EEG detected LVO-a stroke with a high diagnostic accuracy in a population of suspected stroke patients. However, EEG data were of insufficient quality for analysis in around one-third of patients.

In ELECTRA-STROKE, an ambulatory LVO-a stroke detection device was tested by ambulance personnel in the prehospital setting. In line with other prehospital stroke trials,^34,35^ we demonstrate that successful conduct of a prehospital stroke study is feasible but has its challenges. Consistent with the MR ASAP trial,^35^ obtaining written deferred consent proved to be more challenging in a prehospital setting than in stroke trials that ran in an in-hospital setting, such as the MR CLEAN NO-IV trial.^36^ Lack of written informed consent led to exclusion of 17% of the patients. Still, without a deferred informed consent procedure, performing the study would have been logistically more challenging and could have resulted in substantial treatment delays.

Prehospital detection of patients with an LVO-a stroke would enable direct transport of these patients to a comprehensive stroke center while still allowing other suspected stroke patients to be transported to the closest primary stroke center. Data from nonrandomized studies suggest that this workflow can lead to shorter EVT treatment initiation times and better clinical outcomes for patients with an LVO-a stroke.^5,7,9,10,11^ By contrast, the RACECAT trial—performed in a nonurban area with relatively long initial transport times—did not find a difference in clinical outcome in suspected LVO stroke patients between a mothership and drip-and-ship model.^37^ This discrepancy between studies may be attributed to geographic differences and emphasize the need for regional solutions to optimize stroke care.

Recent in-hospital studies have demonstrated the potential of EEG for LVO-a stroke detection. In the first 100 patients enrolled in the in-hospital phase of the ELECTRA-STROKE study, the diagnostic accuracies of the frequency band powers were comparable with this study, with the theta/alpha ratio as the best performing EEG feature (AUC = 0.83).^26^ The pairwise derived Brain Symmetry Index, however, did not have a good diagnostic accuracy in that study (AUC = 0.38), while in this study, this EEG feature showed the best performance (AUC = 0.91). This disparity may be due to a difference in EEG data quality, as the pairwise derived Brain Symmetry Index is known to be highly susceptible to artifacts and this study included an improved, more sensitive, artifact detection algorithm than the in-hospital study. Erani et al.^24^ performed dry electrode EEG recordings in 100 suspected stroke patients and found moderately high diagnostic accuracy of EEG for LVO-a stroke detection (AUC = 0.69) when using a combination of the ipsilesional relative theta and alpha power. We assume that the diagnostic accuracy is lower compared with our study because none of the utilized EEG features contained information on both hemispheres—as is the case for the pairwise derived Brain Symmetry Index—and the diagnostic accuracy was validated on unseen data. In our study, we did not validate the results in unseen data, which may have led to an overestimation of the diagnostic accuracy. Sergot et al.^25^ investigated LVO detection by means of a portable wet electrode EEG and somatosensory-evoked potentials device. They used data of 109 suspected stroke patients and found a sensitivity and specificity for LVO stroke of both 80%.

The value of using prehospital stroke scales, especially the RACE, G-FAST, and ACT-FAST, for LVO-a stroke detection has recently been demonstrated.^15,38^ The best performing scale, the RACE, reached an AUC of 0.83 in a group of 1,039 suspected stroke patients. Although clinical scales have the potential to be easily implemented in the prehospital setting and can be applied to most patients, assessment remains subjective. An important aspect of progress may lie in combining EEG with clinical scales in future studies. The improvement of diagnostic accuracy for LVO-a stroke detection, when combining EEG and clinical data, has been shown by Erani et al.^24^ Unfortunately, both the RACE and G-FAST scales are not routinely used by ambulance personnel in the regions where ELECTRA-STROKE was performed; thus, we could not study the value of combining both techniques in this study.

Our study has several limitations. First, EEG data quality was insufficient in approximately one-third of patients. The fact that ambulance personnel had to perform the measurement in a limited timeframe and while under pressure of transporting a suspected stroke patient as quickly as possible undoubtedly contributed to this. Future innovations in EEG hardware and software should specifically address this issue, especially among groups with higher dropout rates due to insufficient EEG data quality (women, those with longer hair and those with more severe neurologic deficits). Second, we only had a limited number of LVO-a stroke patients in the study, increasing the statistical uncertainty of the results. Third, we had insufficient power to determine whether EEG can distinguish between LVO-a stroke and hemorrhagic stroke. This should be the focus of future studies because hemorrhagic stroke is an important mimic of LVO-a stroke and there are data that suggest that subjecting patients with a hemorrhagic stroke to longer transportation times may negatively affect their outcome.^37^

In conclusion, the data from this study suggest that dry electrode EEG has potential to detect LVO-a stroke among patients with suspected stroke in the prehospital setting. Toward future implementation of EEG in prehospital stroke care, EEG data quality needs to be improved and additional validation in a larger cohort of patients is necessary.

## Appendix Authors

**Table:**

Name	Location	Contribution
Maritta N. van Stigt, MSc	Departments of Clinical Neurophysiology and Neurology, Amsterdam UMC location University of Amsterdam, the Netherlands	Drafting/revision of the manuscript for content, including medical writing for content; major role in the acquisition of data; study concept or design; analysis or interpretation of data
Eva A. Groenendijk, MSc	Departments of Clinical Neurophysiology and Neurology, Amsterdam UMC location University of Amsterdam, the Netherlands	Drafting/revision of the manuscript for content, including medical writing for content; analysis or interpretation of data
Laura C.C. van Meenen, MD, PhD	Department of Neurology, Amsterdam UMC location University of Amsterdam, the Netherlands	Drafting/revision of the manuscript for content, including medical writing for content; major role in the acquisition of data; study concept or design
Anita A.G.A. van de Munckhof, MD, MSc	Department of Neurology, Amsterdam UMC location University of Amsterdam, the Netherlands	Drafting/revision of the manuscript for content, including medical writing for content; major role in the acquisition of data
Monique Theunissen, BSc	Witte Kruis Ambulancezorg, Alkmaar, the Netherlands	Drafting/revision of the manuscript for content, including medical writing for content; major role in the acquisition of data
Gaby Franschman, MD, PhD	Witte Kruis Ambulancezorg, Alkmaar, the Netherlands	Drafting/revision of the manuscript for content, including medical writing for content
Martin D. Smeekes, MD, MSc	Ambulancezorg Nederland, Zwolle, the Netherlands	Drafting/revision of the manuscript for content, including medical writing for content
Joffry A.F. van Grondelle, BSc	Ambulance Amsterdam, the Netherlands	Major role in the acquisition of data
Geertje Geuzebroek, BSc	Ambulance Amsterdam, the Netherlands	Major role in the acquisition of data
Arjen Siegers, MD, MSc	Ambulance Amsterdam, the Netherlands	Drafting/revision of the manuscript for content, including medical writing for content
Marieke C. Visser, MD, PhD	Department of Neurology, Amsterdam UMC location University of Amsterdam, the Netherlands	Drafting/revision of the manuscript for content, including medical writing for content
Sander M. van Schaik, MD, PhD	Department of Neurology, OLVG Hospital location West, Amsterdam, the Netherlands	Drafting/revision of the manuscript for content, including medical writing for content
Patricia H.A. Halkes, MD, PhD	Department of Neurology, Noordwest Ziekenhuisgroep location Alkmaar, the Netherlands	Drafting/revision of the manuscript for content, including medical writing for content
Charles B.L.M. Majoie, MD, PhD	Department of Radiology and Nuclear Medicine, Amsterdam UMC location University of Amsterdam, the Netherlands	Drafting/revision of the manuscript for content, including medical writing for content
Yvo B.W.E.M. Roos, MD, PhD	Department of Neurology, Amsterdam UMC location University of Amsterdam, the Netherlands	Drafting/revision of the manuscript for content, including medical writing for content
Johannes H.T.M. Koelman, MD, PhD	Department of Clinical Neurophysiology, Amsterdam UMC location University of Amsterdam, the Netherlands	Drafting/revision of the manuscript for content, including medical writing for content; study concept or design
Miou S. Koopman, MD, MSc	Department of Radiology and Nuclear Medicine, Amsterdam UMC location University of Amsterdam, the Netherlands	Drafting/revision of the manuscript for content, including medical writing for content; analysis or interpretation of data
Henk A. Marquering, PhD	Department of Radiology and Nuclear Medicine; Department of Biomedical Engineering and Physics, Amsterdam UMC location University of Amsterdam, the Netherlands	Drafting/revision of the manuscript for content, including medical writing for content; study concept or design
Wouter V. Potters, PhD	TrianecT, Utrecht, the Netherlands	Drafting/revision of the manuscript for content, including medical writing for content; study concept or design; analysis or interpretation of data
Jonathan M. Coutinho, MD, PhD	Department of Neurology, Amsterdam UMC location University of Amsterdam, the Netherlands	Drafting/revision of the manuscript for content, including medical writing for content; study concept or design; analysis or interpretation of data

## Glossary

AUC: area under the ROC curve
EVT: endovascular thrombectomy
IQR: interquartile range
LVO-a stroke: anterior circulation large vessel occlusion stroke
ROC: receiver operating characteristic
